# Peptide HER2(776–788) represents a naturally processed broad MHC class II-restricted T cell epitope

**DOI:** 10.1054/bjoc.2001.2089

**Published:** 2001-11

**Authors:** R Sotiriadou, S A Perez, A D Gritzapis, P A Sotiropoulou, H Echner, S Heinzel, A Mamalaki, G Pawelec, W Voelter, C N Baxevanis, M Papamichail

**Affiliations:** Cancer Immunology Immunotherapy Center, Saint Savas Cancer Hospital, Athens, 11522, Greece; Abteilung fur Physikalishe Biochemie des Physiologisch-chemisches, Institut der Universitat Tuebingen, Tuebingen, D 72076; Tuebingen Ageing and Tumor Immunology Group (TATI), Section of Transplantation Immunology, University of Tuebingen Medical School, Tuebingen, D 72072, Germany; Hanson Centre for Cancer Research, Institute of Medical and Veterinary Science, Adelaide, SA, 5000, Australia; Department of Biochemistry, Hellenic Pasteur Institute, Athens, 11521, Greece

**Keywords:** HER-2/neu peptide, CD4^+^ T cell clones, MHC alleles, vaccines, cancer immunotherapy

## Abstract

HER2/neu-derived peptides inducing MHC class II-restricted CD4+ T helper lymphocyte (Th) responses, although critical for tumour rejection, are not thoroughly characterized. Here, we report the generation and characterization of CD4+ T cell clones specifically recognizing a HER-2/neu-derived peptide (776–788) [designated HER2(776–788)]. Such clones yielded specific proliferative and cytokine [gamma-interferon(IFN)-γ] responses when challenged with autologous dendritic cells (DCs) loaded with HER2(776–788). By performing blocking studies with monoclonal antibodies (MAbs) and by using DCs from allogeneic donors sharing certain HLA-DR alleles, we found that HER2(776–788) is a promiscuous peptide presented, at least, by DRB5*0101, DRB1*0701 and DRB1*0405 alleles. One TCRVbeta6.7+ clone recognized the HLA-DRB5*0101+ FM3 melanoma cell line transfected with a full length HER-2/neu cDNA. Moreover, this clone recognized the HER-2/neu+ SKBR3 breast cancer cell line induced to express HLA-DR, thus demonstrating that HER2(776–788) represents a naturally processed and presented epitope. Our data demonstrate that helper peptide HER2(776–788) represents a promiscuous epitope binding to at least three HLA-DR alleles, thus offering a broad population coverage. The use of antigenic peptides presented by major histocompatibility complex (MHC) class II in addition to those presented by class I may improve the therapeutic efficacy of active immunization.© 2001 Cancer Research Campaign  http://www.bjcancer.com


*British Journal of Cancer* (2001) **85**(10), 1527–1534
© 2001 Cancer Research Campaign
doi: 10.1054/ bjoc.2001.2089, available online at http://www.idealibrary.com on IDEAL
http://www.bjcancer.com

### Peptide HER2(776–788) represents a naturally processed broad MHC class II-restricted T cell epitope

**R Sotiriadou[1], SA Perez[1], AD Gritzapis[1], PA Sotiropoulou[1], H Echner[2], S Heinzel[3,4], A Mamalaki[5], G Pawelec[3], W Voelter[2], CN Baxevanis[1] and M Papamichail[1]**

[1]Cancer Immunology Immunotherapy Center, Saint Savas Cancer Hospital, Athens 11522, Greece; [2]Abteilung fur Physikalishe Biochemie des Physiologisch-chemisches Institut der Universitat Tuebingen, Tuebingen D 72076; [3]Tuebingen Ageing and Tumor Immunology Group (TATI), Section of Transplantation Immunology, University of Tuebingen Medical School, Tuebingen D 72072, Germany; [4]Current address: Hanson Centre for Cancer Research, Institute of Medical and Veterinary Science, Adelaide SA 5000, Australia; [5]Department of Biochemistry, Hellenic Pasteur Institute, Athens 11521, Greece

**Summary** HER2/neu-derived peptides inducing MHC class II-restricted CD4+ T helper lymphocyte (Th) responses, although critical for tumour rejection, are not thoroughly characterized. Here, we report the generation and characterization of CD4+ T cell clones specifically recognizing a HER-2/neu-derived peptide (776–788) [designated HER2(776–788)]. Such clones yielded specific proliferative and cytokine [gamma-interferon(IFN)-γ] responses when challenged with autologous dendritic cells (DCs) loaded with HER2(776–788). By performing blocking studies with monoclonal antibodies (MAbs) and by using DCs from allogeneic donors sharing certain HLA-DR alleles, we found that HER2(776–788) is a promiscuous peptide presented, at least, by DRB5*0101, DRB1*0701 and DRB1*0405 alleles. One TCRVbeta6.7+ clone recognized the HLA-DRB5*0101+ FM3 melanoma cell line transfected with a full length HER-2/neu cDNA. Moreover, this clone recognized the HER-2/neu+ SKBR3 breast cancer cell line induced to express HLA-DR, thus demonstrating that HER2(776–788) represents a naturally processed and presented epitope. Our data demonstrate that helper peptide HER2(776–788) represents a promiscuous epitope binding to at least three HLA-DR alleles, thus offering a broad population coverage. The use of antigenic peptides presented by major histocompatibility complex (MHC) class II in addition to those presented by class I may improve the therapeutic efficacy of active immunization. © 2001 Cancer Research Campaign http://www.bjcancer.com

**Keywords**: HER-2/neu peptide; CD4+ T cell clones; MHC alleles; vaccines; cancer immunotherapy

Increasing evidence from both murine and human studies has indicated that optimal antitumour responses require the participation of both CD4+ Th and CD8+ cytotoxic T lymphocyte (CTL) (Pardoll and Topalian, 1998). Originally it was thought that the importance of Th cells was due to production of cytokines, particularly interleukin (IL)-2, which provided growth and differentiation signals for precursor CTL to become effector CTL lysing the autologous tumour (Simpson and Gordon, 1977; Keene and Forman, 1982). More recent reports from several laboratories including our own, however, have ascribed another function to CD4+ T lymphocytes, namely the activation of antigen presenting cells (APCs) so that they can effectively stimulate precursor CTL to become effector CTL (Ridge et al, 1998; Schoenberger et al, 1998; Baxevanis et al, 2000). Recognition of the essential role of CD4+ T lymphocytes for developing effective antitumour responses has been accompanied by the identification of antigens (Ags) on human tumours. Several MHC class I-restricted peptide epitopes are derived from tumour Ags such as tyrosinase, gp100, Melan-A, MART-1, MAGE-3 and NY-ESO-1 which have also been demonstrated also to contain MHC class II-restricted epitopes recognized by CD4+ T lymphocytes (Wang and Rosenberg, 1999; Jaeger et al, 2000).

The HER-2/neu proto-oncogene encodes a transmembrane glycoprotein with extensive homology to epidermal growth factor receptor whose cytoplasmic domain has tyrosine kinase activity (Hung and Lau, 1999). Over-expression of HER-2/neu on many adenocarcinomas of breast and ovary is correlated with poor prognosis (Schultz and Weber, 1999). In the past few years several peptide epitopes from HER-2/neu have been clearly demonstrated to be recognized by ovarian (Ioannides et al, 1991; Peoples et al, 1994; Yoshino et al, 1994; Fisk et al, 1995; Rongcun et al, 1999) and breast (Linehan et al, 1995; Peoples et al, 1995) cancer specific CTL. In addition to the MHC class I-restricted epitopes, there is growing evidence that HER-2/neu protein also contains helper epitopes. Thus, it has already been shown that: (i) some patients with HER-2/neu positive cancers produce IgG antibodies against the HER-2/neu protein (Disis et al, 1994) and (ii) $CD4^+$ T cells from HER-2/neu-positive cancer patients can proliferate and produce cytokines in response to synthetic peptides or recombinant HER-2/neu protein (Disis et al, 1997; Fisk et al, 1997; Tuttle et al, 1998). It is expected that using CTL and Th peptide epitopes together in therapeutic vaccines may enhance their effectiveness in clinical trials.

The purpose of this study was to examine the potential use of HER2(776–788) as a helper epitope and the identification of its potential promiscuity (broad MHC restriction), so as to improve the design of vaccination protocols of CTL-based immunotherapy for tumours over-expressing HER-2/neu. We report here that HER2(776–788) is a helper peptide naturally processed and specifically recognized on tumour cells by CD4+ T cell clones in vitro. HER2(776–788)-activated $CD4^+$ T cells secrete large amounts of IFN-γ, but not IL-4 or IL-10, which is suggestive of a Th1 function of such cells stimulated by this peptide. One of the clones recognized HER2(776–788) in the context of either

*Received 9 April 2001*
*Revised 1 August 2001*
*Accepted 7 August 2001*

*Correspondence to:* CN Baxevanis

HLA-DRB1*0405, DRB1*0701 and DRB5*0101 indicating a high degree of promiscuity both in peptide binding to the class II molecule, and in recognition of different MHC/peptide complexes by the same TCR. These results may encourage the use of the HER2(776–788) peptide along with HER-2/neu-derived CTL epitopes in vaccination protocols for cancer patients of different MHC types whose tumours over-express HER-2/neu.

### MATERIALS AND METHODS

### Cell lines

The human breast adenocarcinoma cell line SKBR3 (American Type Culture Collection, Manassas, VA) was cultured in McCoy's 5A medium supplemented with 10% fetal bovine serum (FBS), 2mM L-glutamine and 50 μg/ml gentamicin (all purchased from Life Technologies, Gaithersburg, MD). SKBR3 cells were induced to express HLA-DR molecules after a 4-day incubation with 150 U/ml human recombinant IFN-γ (Boehringer-Mannheim, Mannheim, Germany) and human recombinant tumour necrosis factor (TNF)-α 100U/ml (R&D System, Abingdon, UK) in a $CO_2$ incubator. The human melanoma cell line FM3 (a kind gift of Dr J Zeuthen, Dept of Tumor Cell Biology, Danish Cancer Society Research Center, Copenhagen) was grown in RPMI 1640 (Life Technologies) with 10% FBS 2mM L-glutamine and 50 μg/ml gentamicin (complete medium).

### Purification of CD4$^+$ T cells

CD4$^+$ T cells were isolated from PBMCs collected by standard procedures from a healthy volunteer [HLA serotyping: HLA-A2, A3, B44, B47, Cw6, DR15(2), DR4, DR51, DR53, DQ6(1), DQ8(3)].

Highly purified CD4$^+$ T cells (>98% purity) were isolated using Dynabeads (CD4 Positive Isolation Kit; Dynal, Oslo, Norway), as recently described (Baxevanis et al, 1999).

### Generation of dendritic cells

DCs autologous to the CD4$^+$ T cells were generated from the adherent fraction of freshly isolated PBMCs. Briefly, $4 \times 10^6$ PBMCs in 2 ml X-VIVO 15 (BioWhittaker, Walkersville, MD) were plated in 6-well plates (Costar, Corning Inc., NY) and incubated for 2 h in a $CO_2$ incubator. Non adherent cells were gently washed out with Hanks' Balanced Salt Solution (HBSS; Life Technologies). The remaining plastic adherent cells were cultured in 2 ml X-VIVO 15 medium supplemented with 1000 IU/ml IL-4 (R&D Systems, Europe) and 1000 IU/ml granulocyte macrophage-colony stimulating factor (GM-CSF) (Immunex, Seattle, WA). Fresh medium (2 ml) and cytokines were added on days 2 and 4. For induction of maturation, TNF-α was added at 10 ng/ml on day 6. The nonadherent cell population was used on day 7 as a source of enriched mature dendritic cells or cryopreserved for later use. The percentage of mature DCs recorded was > 50%, as based on the CD3–, CD14–, CD16–, CD20–, CD40+, CD80+, CD83+, CD86+, and HLA-DR+ phenotype analysed by flow cytometry. Mature DCs were used as APCs pretreated with 70 μg/ml mitomycin C (Kyowa, Tokyo, Japan) for 45 min at 37˚C. After extensive wash with HBSS, DCs were pulsed with the appropriate concentration of peptide for 4 h at 37˚C.

HLA alleles presented in Table 1 were determined by standard genotyping methods. Alleles HLA-DRB1*0401, *0404 and *0405 correspond to DR4, DRB1*1501 to DR15(2), DRB1*1101, 1104 to DR11(5), DRB*1601 to DR16(2), DRB1*0301 to DR17(3), DRB1*1001 to DR10, DRB1*1401 to DR14(6), DRB1*0701 to DR7, DRB5*0101 to DR51, DRB4*0101 to DR53, DRB3*0101 and *0201 to DR52.

### Peptide synthesis

Synthesis of the HER-2(776–788) (GSPYVSRLLGICL) peptide and of control peptides HER-2(884–899) (VPIKWMALE-SILRRRF) (Fisk et al, 1997), gp100(44–59) (WNRQ-LYPEWTEAQRLD) (Halder et al, 1997) and tyrosinase(448–462) (DYSYLQDSDPDSFQD) (Topalian et al, 1996) was performed with an Ecosyn P peptide synthesizer (Eppendorf-Biotronik, Hamburg, Germany), using the Fmoc strategy and a 4-alcoxy-benzyl alcohol resin. The peptides were assayed for purity by analytical HPLC, amino acid analysis and matrix-assisted laser desorption mass spectrometry. All peptides were determined to be > 95% pure. Peptides were lyophilized, dissolved in

**Table 1**

| Donor (No.) | HLA alleles | SI[a] |
|---|---|---|
| 1[b] | DRB1*1501, 0404, DRB5*0101, DRB4*0101 | 2.9 |
| 2 | DRB1*1501, 1101, DRB5*0101, DRB3*0101 | 4.7 |
| 3 | DRB1*1601, DRB5*0101 | 3.9 |
| FM3-HER[c] | DRB1*0201, 0401, DRB5*0101, DR53[d] | 3.2 |
| 5 | DRB1*0301, DRB3*0201 | 1.1 |
| 6 | DRB1*0404, 0301, DRB3*0101, DRB4*0101 | 1 |
| 7 | DRB1*0404, 1104, DRB3*0201, DRB4*0101 | 1.2 |
| 8 | DRB1*1001, 1104, DRB3*0101 | 1.4 |
| 9 | DRB1*0401, 1401, DRB3*0101 | 1.3 |
| 10 | DRB1*0401, 1104, DRB3*0201, DRB4*0101 | 1.2 |
| 11 | DRB1*0405, 0404, DRB4*0101 | 3.3 |
| 12 | DRB1*0405, 1104, DRB3*0201, DRB4*0101 | 2.6 |
| 13 | DRB1*0701, 1104, DRB3*0201, DRB4*0101 | 2.8 |
| 14 | DRB1*0701, 1104, DRB3*0201, DRB4*0101 | 2.6 |
| SKBR3[e] | DRB1*0701, 1302, DR52[d], DR53[d] | 3.1 |

[a]DCs from the healthy donors listed above were used as APCs for HER2(776–788) presentation to clone 2.1. [b]Clone 2.1 was generated from PBMCs of donor No 1. [c]FM3 transfected with a HER-2/neu cDNA. [d]Only serological typing was performed. [e]SKBR3 were treated with rIFN-γ +rTNF-α.

 *© 2001 Cancer Research Campaign*

phosphate-buffered saline (PBS), aliquoted at 2 mg/ml and stored frozen at –20˚C until use (Reicher et al, 1996).

### Monoclonal antibodies and immunophenotyping

MAbs to TCR α/β common and variable regions: Vβ3.1, Vβ5(a), Vβ6.7, Vβ7.1, Vβ8(a), Vβ12, Vβ13 and Vβ17 conjugated with FITC were obtained from Endogen (Boston, MA). Anti-CD83 conjugated with PE MAb was obtained from Caltag Laboratories (Burlingame, CA). Anti-TCR Vβ9 conjugated with PE and anti-TCR Vβ11, Vβ5.1, Vβ21, Vβ13.6, Vβ7, Vβ22, Vβ16, Vβ14, Vβ20, Vβ2 conjugated with FITC were purchased from Serotec Ltd (Oxford, UK). Isotype matched anti-IgG conjugated with FITC ($IgG_1$, clone G18–145) was purchased from Becton Dickinson (Mountain View, CA). All other MAbs were obtained from PharMingen (San Diego, CA). Anti-CD4, -CD8, -CD16, -CD20, -CD40 and anti-CD80 were conjugated with FITC. Anti-CD3, -CD14, -CD56, -CD86, -HLA-DR and anti-TCR Vβ23 MAbs were conjugated with PE. Samples were analysed using FACScan (Becton Dickinson) and CellQuest analysis software. For blocking experiments, purified azide-free anti-HLA-DR (clone L243, IgG2a, κ), anti-TNP, (clone G155–178, IgG2a, κ) (both from PharMingen), HLA-DP (clone B7/21, Becton Dickinson) and anti-HLA-A,B,C (clone W6/32) and anti-HLA-DQ (clone SPVL3) (both from Serotec) were used.

### Generation of CD4+ T cell line and clones specific for HER2(776–788)

Highly purified CD4+ T cells were plated in 96-well U-bottom plates (Costar) at $5 \times 10^4$ cells/well with $10^4$ well autologous mature DCs were pulsed with 25 μg/ml HER-2 (776–788) peptide. Cultures were set up in a final volume of 200 μl X-VIVO 15 medium supplemented with 1% autologous heat-inactivated plasma, 10 ng/ml IL-7 (R&D systems) and 100 pg/ml IL-12 (R&D systems) in a $CO_2$ incubator. Growing cultures were restimulated at weekly intervals with DCs pulsed with 5 μg/ml of the peptide. After 3 days from the first restimulation, IL-2 (Chiron Corporation, California) was added at 10 IU/ml. Peptide-specific T cell clones were obtained from this bulk culture by limiting dilution. Cloning was accomplished in X-VIVO 15 medium supplemented with 0.5 μg/ml PHA (Sigma), 1 IU/ml IL-2, 10 ng/ml IL-7, 100 pg/ml IL-12 and mitomycin C treated allogeneic PBMCs as feeders ($2 \times 10^4$/well).

### Transfection of the FM3 cell line

FM3 cells were co-transfected with a pSV2-c-erbB2 construct (kindly provided by Dr Mien-Chie Hung, MD Anderson Cancer Ctr, Houston, Texas) and a pSV2neo plasmid using DNA-calcium phosphate co-precipitates as described (Graham and van der Eb, 1973). Selection with G-418 (500 μg/ml) was started 48 h later. For cloning the cells were distributed into 96-well flat bottom tissue culture plates (Costar) and stable transfectants were selected. HER-2/neu-transfected FM3 lines were subcloned until all cells were found to express HER-2/neu (thereafter referred to as FM3/HER) as detected by FACS using FITC-conjugated anti-HER-2/neu MAb (clone Neu 24.7) recognizing the extracellular domain of HER-2/neu (Becton Dickinson).

### Proliferation assay

Responder $CD4^+$ T cells ($2\times10^4$ cells/well) were co-cultured with peptide (5 μg/ml) pulsed or unpulsed autologous DCs ($2\times10^3$ cells/well) as APCs in 96-well U-bottom plates in a final volume of 200 μl X-VIVO 15 medium supplemented with 10 ng/ml IL-7 and 100 pg/ml IL-12. FM3 or FM3/HER cells and SKBR3 cells (non-treated or treated with IFN-γ plus TNF-α) were also used as APCs at $2 \times 10^3$ cells/well. MAbs, wherever indicated, were added at 10 μg/ml final concentration throughout the culture period. On day 4, 1 μCi/well tritiated thymidine ([$^3$H]TdR, 48.0 Ci/mmol, 1mCi/ml, Amersham Pharmacia Biotech, Buckinghamshire, UK) was added for the last 16 h. Cells were then harvested and [$^3$H]TdR incorporation was measured in a liquid scintillation counter (LKB Wallac, Turku, Finland). All cultures were performed in triplicates and results are expressed as counts per minute (cpm). Stimulation index (SI) is calculated with cpm obtained in the presence of antigen (i.e peptide-pulsed DCs, FM3/HER or treated SKBR3 cells) divided by cpm obtained in the absence of antigen (i.e. unpulsed DCs, wild-type FM3 or untreated SKBR3 cells).

### ELISPOT assay

96-well flat-bottomed Immobilon-P PVDF plates (Multi-Screen-IP, Millipore, Bedford, MA) were coated overnight at 4˚C with 2 μg/ml anti-IFN-γ MAb (NIB42, PharMingen). Responder T cells were cultured in the 96-well nitrocellulose pre-coated plates at $2\times10^4$ cells/well with autologous peptide (5 μg/ml) pulsed or unpulsed DCs at $2\times10^3$ cells/well in 200 μl X-VIVO 15 medium supplemented with 1 IU/ml IL-2, 10ng/ml IL-7 and 100 pg/ml IL-12 for 24 h at 37˚C. After incubation, the plates were washed extensively (with a solution of 0.05% Tween 20/PBS) and supplemented with the biotinylated anti-IFN-γ MAb (4S.B3, PharMingen). Plates were incubated for 2 h at room temperature, washed and developed with alkaline phosphatase-conjugated streptavidin (Bio-Rad Laboratories, Hercules, CA) for another 1 h. After washing, BCIP/NTB substrate (Bio-Rad) was used to develop dark-violet spots. Spots were counted under a stereomicroscope (Zeiss, Germany). % inhibition of IFN-γ spots by the HLA-DR MAb was calculated as follows:

$$\frac{\text{no. of spots w/o MAb} - \text{no. of spots with MAb}}{\text{no. of spots w/o MAb}} \times 100$$

SI was calculated with the number of spots with peptide-pulsed DCs divided by the number of spots with non-pulsed DCs.

### Quantitation of cytokines in culture supernatants

IFN-γ, IL-4 and IL-10 secretion by HER2(776–788) specific $CD4^+$ T cell clone 2.1 was estimated with commercially available ELISA Kits (Diaclone Research, Besançon, France) according to the manufacturer's instructions.

### RESULTS

### Generation of a CD4+ T cell line specifically recognizing HER2(776–788)

A CD4+ T cell line from a healthy volunteer was established by stimulation with HER2(776–788)-pulsed DC and was shown to be specific for HER2(776–788) in proliferation assays. A strong

© 2001 Cancer Research Campaign response was detected in the presence of autologous DCs pulsed with this peptide which was to a great extent blocked with an anti-HLA-DR (MHC class II) MAb, but not with an isotype matched control MAb or an anti-HLA-A,B,C (MHC class I) MAb (Figure 1). MAb recognizing monomorphic determinants on MHC class II molecules DP and DQ remained also without any effect (Figure 1). The specificity of this CD4+ T cell line was further tested against autologous DCs pulsed with irrelevant peptides from HER-2/neu [HER2(884–899)], gp100 [gp(44–59)] or tyrosinase [tyro(448–462)] all of which have been reported to be recognized by CD4+ T cells in the context of HLA-DR molecules (Topalian et al, 1996; Halder et al, 1997; Kobayashi et al, 2000). As shown in Figure 2, none of these could significantly stimulate proliferative response in the HER2(776–788) specific CD4+ T cell line. The same line was unable to recognize the HLA-DR+, HER-2/neu negative(–) FM3 melanoma cell line. However, it proliferated strongly upon stimulation with HER-2/neu-transfected FM3 cells (FM3/HER) and this response was also substantially blocked in the presence of the anti-HLA-DR MAb (Figure 2). These data suggested that the CD4+ T line specifically recognizes the HER-2/neu peptide. Moreover, the significant recognition of FM3/HER cells suggests that HER2(776–788) represents a naturally processed and presented epitope.

### Generation and characterization of HER2(776–788)-specific CD4+ T cell clones

To better characterize the HER-2/neu peptide-specific response, CD4+ T cell clones were generated. The CD4+ T line was cloned by limiting dilution using allogeneic PBMCs as feeders. Of the clones generated, eight proliferated in response to HER2(776–788)-pulsed autologous DCs with a SI greater than 1.5 (range: 1.5–4.9) (Figure 3). Moreover, all clones could recognize the FM3/HER, but not the wild-type FM3 cells (SI range:1.7–4.9) (Figure 3). HER2(776–788) peptide recognition was confirmed by using ELISPOT assays. The same clones which significantly proliferated in response to autologous DCs pulsed with HER2(776–788) produced significant numbers of IFN-γ spots when similarly stimulated (Figure 4). The SI observed in these experiments ranged between 1.8 and 7.7 IFN-γ spots were considered significant when the difference between the mean number from each peptide-stimulated culture (every clone was tested in duplicates) was higher than the mean number from all unstimulated cultures (mean value ± SD from eight cultures:33 ± 7) plus 2SD (i.e. > 47), which was the case in all clones tested. Similar to their parental CD4+ T-cell line, all clones recognized HER2(776–788) in the context of HLA-DR since their production of IFN-γ spots was greatly inhibited when an anti-HLA-DR MAb was added at culture initiation (range of inhibition:46–60%).

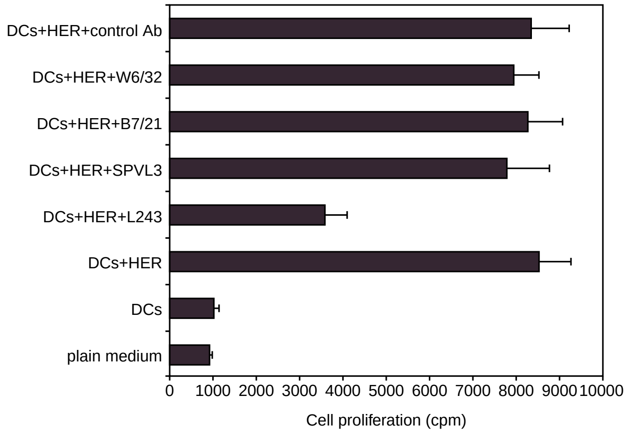

**Figure 1** HER2(776–788) sensitized CD4+ T cells recognize in an HLA-DR restricted fashion autologous DCs loaded with the same peptide. CD4+ T cells from a healthy donor were sensitized with HER2(776–788) in long-term cultures as described in 'M+M'. After the 3rd restimulation (day 30) recovered CD4+ T cells were tested as indicated in the proliferation assay. MAbs specific for HLA-DR (L243), HLA-DQ (SPVL3), HLA-DP (B7/21) or HLA-class I (W6/32) were added at 10 μg/ml at culture initiation. Control MAb was an anti-IgG2a,κ isotype matched with L243. Bars represent mean values ± SD from triplicate cultures. One representative experiment out of three conducted is shown

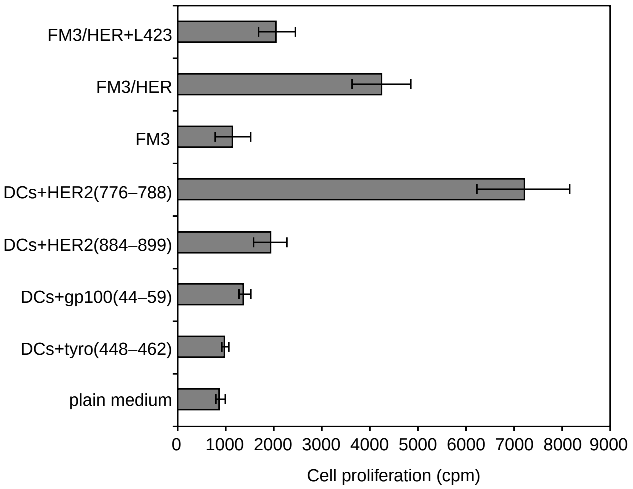

**Figure 2** CD4+ T cells sensitized in vitro with HER2(776–788) specifically recognize this peptide either loaded on DCs or naturally processed and presented by the HLA-DR+ melanoma cell line FM3 transfected to express HER-2/neu (FM3/HER). The same CD4+ T cells were tested as in Figure 1. FM3 melanoma cells were transfected to express HER-2/neu as described in 'M+M'. Bars represent means ± SD from triplicate cultures. One representative experiment out of four conducted is shown

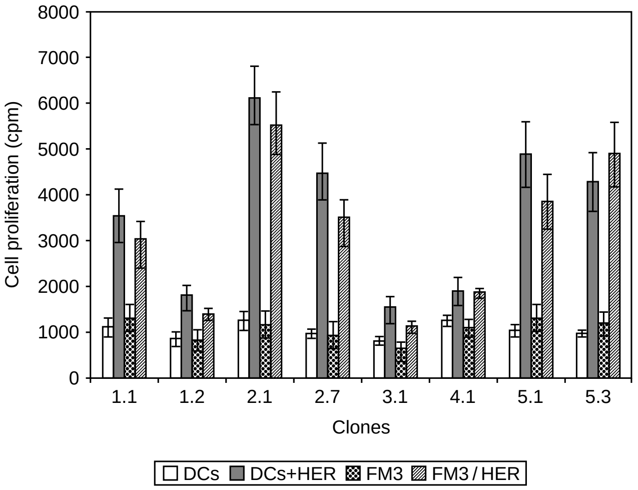

**Figure 3** Recognition of HER2(776–788) by a panel of CD4+ T cell clones. All clones were generated from the CD4+ T cell culture that showed specificity for HER2(776–788) in Figures 1 and 2. Cloning was performed as described in 'M+M'. Each clone was tested twice to ensure reproducibility of the results and bars represent means ± SD from the two independently performed experiments

© 2001 Cancer Research Campaign

### HER2(776–788) recognition by clone 2.1

CD4+ T cell clone 2.1 was propagated in vitro and functionally characterized further. This clone was highly sensitive, recognizing autologous HER2(776–788)-pulsed DCs with a half-maximal proliferation at 0.8 µg/ml of the peptide (Figure 5, filled symbol). Anti-HLA-DR MAb significantly blocked the proliferation at all peptide doses tested, thus proving that there was no mitogenic effect mediated by the peptide at doses higher than 0.5 µg/ml (Figure 5, open symbols). The same clone also recognized the peptide naturally processed and presented by both the FM3/HER melanoma cells and the HER-2/neu+ SKBR3 breast cancer cells induced to express HLA-DR upon preincubation with IFN-γ and TNF-α. This was shown by means of proliferation (Figure 6A) and IFN-γ secretion (Figure 6B). No IL-4 or IL-10 was produced by clone 2.1 in response to FM3/HER and SKBR3 treated cells (data not shown).

Phenotypic analysis of clone 2.1 demonstrated that this was CD4+TCRVB6.7+ (Figure 7). This clone did not express CD8, CD56 or CD16. In addition, when tested with a series of TCRBV MAbs other than anti-TCRVβ6.7 (i.e. Vβ3.1, Vβ5(α), Vβ7.1, Vβ8(α), Vβ12, Vβ13, Vβ17, Vβ11, Vβ5.1, Vβ21, Vβ13.6, Vβ7, Vβ22, Vβ16, Vβ14, Vβ20, Vβ2 and Vβ23) it was also found negative (data not shown).

The data so far suggested that HER2(776–788) is presented in the context of HLA-DR and in particular of the HLA-DRB5*0101 allele which was shared between the FM3 melanoma cell line and the donor of the T cell line (donor 1; Table 1). However, this particular DR-allele was not identified in the SKBR3 cells which express DRB1*0701, 1302, DR52 and DR53 after IFN-γ plus TNF-α-treatment but nonetheless stimulate the clone (Table 1). Therefore, to identify the HLA-DR alleles able to present the peptide, we tested additional DCs from different donors for their capacity to present this particular peptide to clone 2.1. We found that these were capable of presenting the peptide only when expressing one of the alleles: DR5*0101 (donors 1–3), DRB1*0405 (donors 11,12) or DRB1*0701 (donors 13, 14) (SI range: 2.6–4.7) (Table 1). From the same table it can be easily seen that HER2(776–788) was not recognized by clone 2.1 when presented by DRB1*0301 (donors 5,6), DRB1*0401 (donors

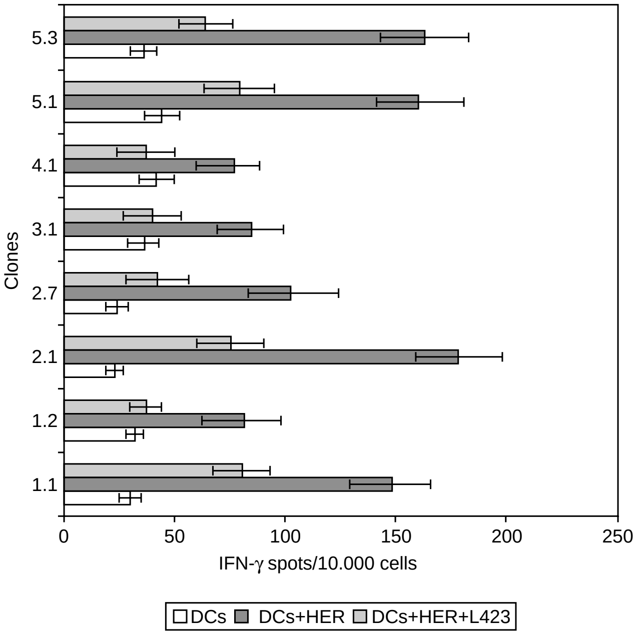

**Figure 4** ELISPOT assays showing recognition of DCs pulsed with HER2(776–788) by autologous CD4+ T cell clones. All clones were tested in 24 h cultures for recognition of autologous DCs either unpulsed or pulsed with HER2(776–788). MAb specific for HLA-DR (L243) was tested for its capacity to inhibit the IFN-γ production. Each clone was tested twice and bars represent means ± SD from the two independently performed experiments

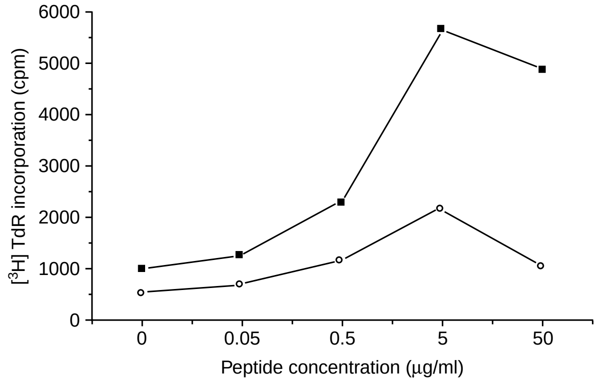

**Figure 5** Titration experiments. DCs pulsed with HER2(776–788) at the indicated concentrations were used to stimulate clone 2.1 in the absence (filled squares) or presence of anti-HLA-DR MAb (open circles). One representative experiment out of three performed is shown. Data are shown as means from triplicate cultures. The SD was negligible (<5% of the means) and thus omitted

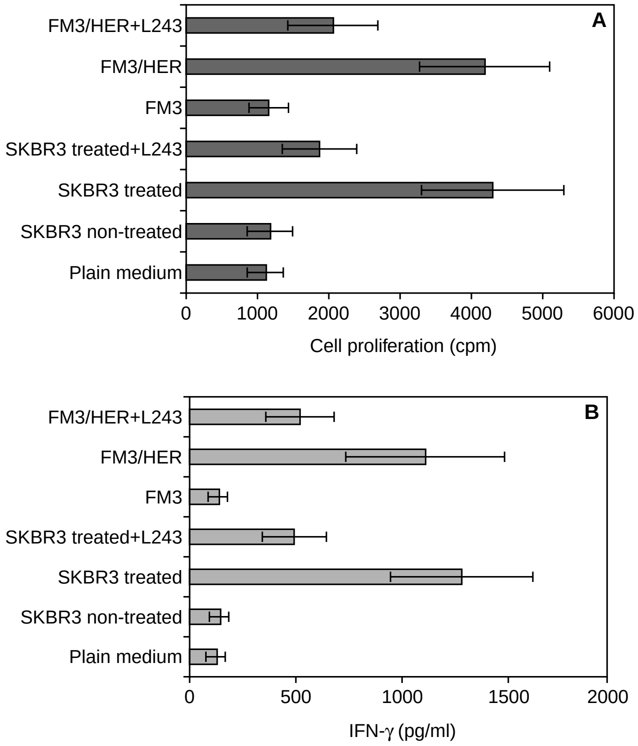

**Figure 6** SKBR3 cells induced to express HLA-DR and HLA-DR+ FM3/HER cells naturally process and present HER2(776–788) to clone 2.1 SKBR3 cells were preincubated with a combination of rIFN-γ plus rTNF-α (SKBR3 treated) as described in 'M+M'. Specific recognition of HER2(776–788) by clone 2.1 was shown either as proliferation (**A**) or as IFN-γ secretion (**B**). Mean values ± SD from three independently performed experiments are shown

*© 2001 Cancer Research Campaign*

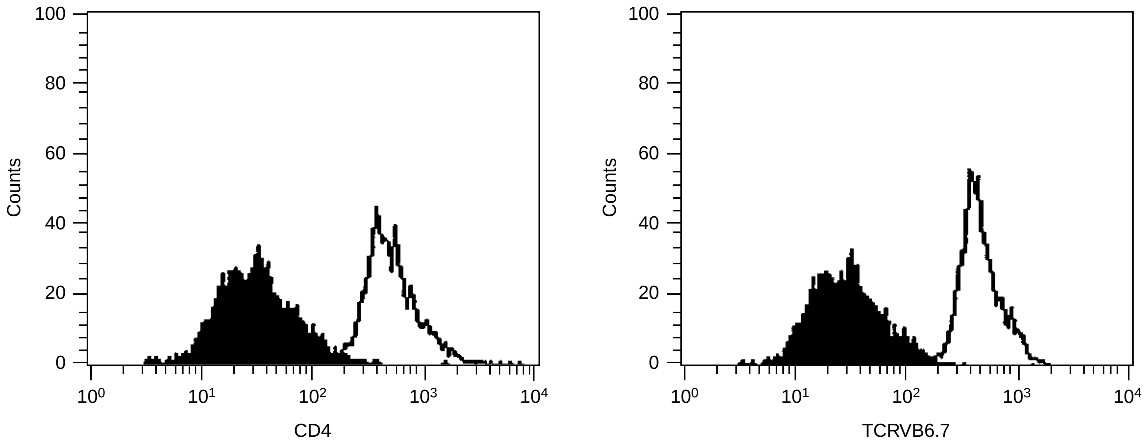

**Figure 7** Staining of HER2(776–788) specific clone 2.1. Cells were incubated with FITC conjugated anti-CD4 (left panel) or anti-TCRVβ6.7 (right panel). Negative control cells were stained with an FITC-conjugated isotype matched MAb

9,10), DRB1*0404 (donors 6,7), DRB1*1001 (donor 8), DRB1*1104 (donor 7, 8, 10), DRB1*1401 (donor 9), DRB3*0101 (donors 6, 8, 9), DRB3*0201 (donors 5,7,10) and DRB4*0101 (donors 6,7,10).

### DISCUSSION

The data presented herein characterize CD4+ T cell responses to HER2(776–788), show direct recognition of this epitope on HER-2/neu+ tumours and provide a rational basis for its use in T-cell based cancer immunotherapy together with MHC class I HER2/neu epitopes. In particular, we showed at the clonal level that HER2(776–788) is capable of specifically activating CD4+ T lymphocytes to strongly proliferate and produce high levels of IFN-γ. By using the FM3 melanoma cell line transfected with HER-2/neu (FM3/HER), as well as the HER2/neu+ SKBR3 breast cancer cell line induced to express HLA class II molecules, we demonstrated that HER2(776–788) represents a naturally processed and presented epitope. Last, we have shown that the same peptide was recognized by a CD4+ T lymphocyte clone in the context of three different HLA-DR alleles, indicating a high degree of histocompatibility and TCR promiscuity.

Our studies show that HER2(776–788) can be recognized by a monoclonal CD4+ T lymphocyte line, clone 2.1, in the context of at least three different HLA-DR alleles, namely DRB5*0101, DRB1*0701 and DRB1*0405. Southwood et al (1998) suggested a motif for promiscuous binding peptides, characterized by a large aromatic or hydrophobic residue in position 1 (p1) and a small, noncharged residue in position 6 (p6). HER2(776–788) fulfils these criteria, as it bears Tyr and Val, either of which could be a potential candidate for p1 and also Leu and Gly that could fit at p6. DRB5*0101 and DRB1*0701 alleles are characterized by largely overlapping peptide-binding repertoires and they belong to a single DR supertype according to Southwood et al (1998). The same peptides also exhibit significant frequencies of cross-reactivity with a group of three other alleles including DRB1*0405 (Southwood et al, 1998). Taken together all these findings satisfactorily explain the binding of HER2(776–788) to the three different DR alleles. Considering also recent data published by Anderson et al (2000) who could induce a response to HER2(776–788) in two donors sharing HLA-DR11 molecule we may ascribe a high degree of promiscuity to this particular peptide. A similar finding has been recently described by Kobayashi et al (2000) which demonstrated recognition of HER2/neu-derived peptide (883–899) presented by HLA-DR1, HLA-DR4, HLA-DR52 and HLA-DR53 molecules.

Despite the high degree of specificity of ligand recognition by TCRs, several reports have described antigen specific T cells that can recognize and respond to diverse MHC molecules (Karr et al, 1991; Hemmer et al, 2000). Doherty et al (1998) have reported the recognition of a herpes simplex virus peptide by a T lymphocyte clone presented in the context of several DR alleles. Hypervariable regions HVR3 of DRβ polypeptides encompassing residues 67–71 and in particular residue 71 have been demonstrated to exert a central role in peptide binding and allorecognition (Coppin et al, 1993; Martinez-Soria et al, 1994; McKinney et al, 1994). In addition, position 86 has also been described to affect TCR allorecognition (Busch et al, 1991; Demotz et al, 1993; Zeliswewski et al, 1993). HLA-DRB5*0101, DRB1*0701 and DRB1*0405 alleles display largely overlapping aminoacid sequences at positions 67–71 and 86 (Table 2) and this may account for the recognition by the same TCR of the peptide complexed to distinct MHC molecules.

The procedure described here proved to be efficient for optimal activation of anti-HER2 (776–788) CD4+ T cell precursors present in the PBMC from our healthy donor, and for the resulting isolation of the peptide-specific CD4+ T lymphocyte clones. Bulk CD4+ T lymphocytes proliferated specifically in an HLA-DR restricted fashion with high stimulation indices in response to autologous DCs pulsed with HER2(776–788). T cell clones established from these cells strongly proliferated and produced high levels of IFN-γ in response to autologous DCs pulsed with the HER2(776–788) peptide, suggesting that under the conditions used HER2(776–788) peptide functions as a Th peptide. Clone 2.1, which could be further propagated, was used to

**Table 2**

| HLA-allele[a] | 67 | 68 | 69 | 70 | 71 | 86 |
|---|---|---|---|---|---|---|
| DRB5*0101 | F | L | E | D | R | G |
| DRB1*0701 | I | L | E | D | R | G |
| DRB1*0405 | L | L | E | Q | R | G |

[a]For more details see ref. (Demotz et al, 1993; McKinney et al, 1994; Doherty et al, 1998).

 © 2001 Cancer Research Campaign confirm the specificity against the naturally processed HER-2/neu-derived peptides. For this, the HLA-DR51+ FM3 melanoma cell line was transfected with a full-length HER-2/neu cDNA (FM3/HER) and specific recognition by this particular clone measured as proliferation and IFN-γ (but not IL-4 or IL-10) secretion was shown. That HER2(776–788) is a naturally processed and presented peptide was confirmed by using the SKBR3 breast cancer cell line, which overexpresses HER-2/neu without enforced expression by transfection. This cell line expresses, in our hands, the HLA-DR7, -13, -52 and -53 alleles upon treatment with a combination of IFN-γ plus TNF-α (Table 1) and exhibits enhanced expression of costimulatory molecules CD80 and CD86 (unpublished data). Clone 2.1 recognized the IFN-γ plus TNF-α-treated SKBR3 cells in a fashion similar to that with the FM3/HER cells (Figure 6), thus confirming that HER2(776–788) is naturally processed and presented on the surface of carcinomas.

This again suggests that HER-2/neu possesses lysosomal targeting sequences that enables it to present its peptides in the context of class II molecules. Indeed, some patients with breast cancer produce anti-HER-2/neu IgG antibodies (Cheever et al, 1995), suggesting that HER-2/neu is presented to CD4+ T lymphocytes in vivo. Since breast tumour cells usually do not express class II molecules, this can be achieved by the uptake of tumour cell apoptotic bodies by macrophages or dendritic cells. The HER-2/neu molecule therefore represents a source of naturally processed and presented peptides capable of stimulating both CD8+ and CD4+ T-lymphocyte responses in vivo. Based on recent data from us (Baxevanis et al, 2000) and others (Ridge et al, 1998; Schoenberger et al, 1998) on CTL-Th collaboration models, it may be hypothesized that HER-2/neu specific CD4+ T lymphocytes can function in vivo as ‘activators’ of APCs so that they can stimulate directly HER-2/neu recognizing CD8+ T lymphocytes to become effector CTLs. Work is now in progress in our laboratory to address this hypothesis.

Taken together the data presented in this report strongly suggest that peptide HER2(776–788) represents a candidate helper epitope to be included in vaccine preparation consisting of HER-2/neu peptides presented by MHC class I and class II molecules. With such formulations vaccine-induced HER-2/neu-specific CD4+ T-lymphocyte responses may synergize with vaccine-induced CTL, resulting in improved antitumour responses. The finding that this peptide is presented in the context of three DR alleles is advantageous since: (i) it may induce higher frequency of clones recognizing it and thus a more massive anti-tumour response; and (ii) it offers a broad population coverage. The identification of tumour protein-derived Th epitopes will be useful for the design of clinical protocols aiming at the improvement of clinical results in cancer immunotherapy.

### ACKNOWLEDGEMENT

Grant sponsor: General Secretary of Research and Technology and Deutches Zentrum fur Luftund Raumfahrt; Grant number: GRI-0606-097; Grant sponsor: Greek General Secretary of Research and Technology; Grant number: 97 EKBAN-19; Grant sponsor: European Commission EUCAPS; Grant number: BMH4-CT98-3058.

### REFERENCES

Anderson BW, Kudelka AP, Honda T, Pollack MS, Gershenson DM, Gillogly MA, Murray JL and Ioannides CG (2000) Induction of determinant spreading and of Th1 responses by in vitro stimulation with HER-2 peptides. *Cancer Immunol Immunother* **49**: 459–468

Baxevanis CN, Spanakos G, Voutsas IF, Gritzapis AD, Tsitsilonis OE, Mamalaki A and Papamichail M (1999) Increased generation of autologous tumor-reactive lymphocytes by anti-CD3 monoclonal antibody and prothymosin α. *Cancer Immunol Immunother* **48**: 71–84

Baxevanis CN, Voutsas IF, Tsitsilonis OE, Gritzapis AD, Sotiriadou R and Papamichail M (2000) Tumor-specific CD4+ T lymphocytes from cancer patients are required for optimal induction of cytotoxic T cells against the autologous tumor. *J Immunol* **164**: 3902–3912

Busch R, Hill CM, Hayball JD, Lamb JR and Rothbard JB (1991) Effect of natural polymorphism at residue 86 of the HLA-DR beta chain on peptide binding. *J Immunol* **147**: 1292–1298

Cheever MA, Disis ML, Bernhard H, Gralow JR, Hand SL, Huseby ES, Qin HL, Takahashi M and Chen W (1995) Immunity to oncogenic proteins. *Immunol Rev* **145**: 33–59

Coppin HL, Carmichael P, Lombardi G, L’Faqihi FE, Salter R, Parham P, Lechler RI and DE Preval C (1993) Position 71 in the alpha helix of the DRbeta domain is predicted to influence peptide binding and plays a central role in allorecognition. *Eur J Immunol* **23**: 343–349

Demotz S, Barbey C, Corradin G, Amoroso A and Lanzavecchia A (1993) The set of naturally processed peptides displayed by DR molecules is tuned by polymorphism of residue 86. *Eur J Immunol* **23**: 425–432

Disis ML, Calenoff E, McLaughlin G, Murphy AE, Chen W, Groner B, Jeschke M, Lydon N, McGlynn E, Livingston RB, Moe R and Cheever MA (1994) Existent T-cells and antibody immunity to HER-2/neu protein in patients with breast cancer. *Cancer Res* **54**: 16–20

Disis ML and Cheever MA (1997) HER-2/neu protein: A target for antigen-specific immunotherapy of human cancer. *Adv Cancer Res* **71**: 342–371

Doherty DG, Penzotti JE, Koelle DM, Kwok WW, Lybrand TP, Masewicz S and Nepom GT (1998) Structural basis of specificity and degeneracy of T cell recognition: pluriallelic restriction of T cell responses to a peptide antigen involves both specific and promiscuous interactions between the T cell receptor, peptide, and HLA-DR. *J Immunol* **161**: 3527–3535

Fisk B, Blevins TL, Wharton JT and Ioannides CG (1995) Identification of an immunodominant peptide of HER-2/neu protooncogene recognized by ovarian tumor-specific cytotoxic T lymphocyte lines. *J Exp Med* **181**: 2109–2117

Fisk B, Hudson JM, Kavanagh J, Wharton JT, Murray JL, Ioannides CG and Kudelka AP (1997) Existent proliferative response of peripheral blood mononuclear cells from healthy donors and ovarian cancer patients to HER-2 peptides. *Anticancer Res* **17**: 45–53

Graham FL and Van der Eb AJ (1973) A new technique for the assay of infectivity of human adenovirus 5 DNA. *Virology* **52**: 456–467

Halder T, Pawelec G, Kirkin AF, Zeuthen J, Meyer HE, Kun L and Kalbacher H (1997) Isolation of novel HLA-DR restricted potential tumor-associated antigens from the melanoma cell line FM3. *Cancer Res* **57**: 3238–3244

Hemmer B, Pinilla C, Gran B, Vergelli M, Ling N, Conlon P, McFarland HF, Houghten R and Martin R (2000) Contribution of individual amino acids within MHC molecule or antigenic peptide to TCR ligand potency. *J Immunol* **164**: 861–871

Hung MC and Lau YK (1999) Basic science of HER-2/neu: a review. *Semin Oncol* **26**: 51–59

Ioannides CG, Freedman RS, Platsoukas CD, Rashed S and Kim YP (1991) Cytotoxic T cell clones isolated from ovarian tumor-infiltrating lymphocytes recognize multiple antigenic epitopes on autologous tumor cells. *J Immunol* **146**: 1700–1707

Jaeger E, Jaeger D, Karbach J, Chen YT, Ritter G, Nagata Y, Gnjatik S, Stockert E, Arand M, Old LJ and Knuth A (2000) Identification of NY-ESO-1 epitopes presented by human histocombatibility antigen (HLA)-DRB4*101–103 and recognized by CD4+ T lymphocytes of patients with NY-ESO-1-expressing melanoma. *J Exp Med* **191**: 625–630

Karr RW, Panina-Bordignon P, Yu WY and Lanzavecchia A (1991) Antigen-specific T cells with monogamous or promiscuous restriction patterns are sensitive to different HLA-DR beta chain substitutions. *J Immunol* **146**: 4242–4247

Keene JA and Forman J (1982) Helper activity is required for the in vivo generation of cytotoxic T lymphocytes. *J Exp Med* **155**: 768–782

Kobayashi H, Wood M, Song Y, Appella E and Celis E (2000) Defining promiscuous MHC class II helper T-cell epitopes for the HER2/neu tumor antigen. *Cancer Res* **60**: 5228–5236

Linehan DC, Goedegebuure PS, Peoples GE, Rogers SO and Eberlein TJ (1995) Tumor-specific and HLA-A2-restricted cytolysis by tumor-associated lymphocytes in human metastatic breast cancer. *J Immunol* **155**: 4486–4491

© 2001 Cancer Research Campaign 

Martínez-Soria E, Steimle V, Burkhardt C, Beffy P, Tiercy JM, Epplen JT, Mach B and Irlé C (1994) An HLA-DRB alpha-helix motif shared by DR11 and DR8 alleles is implicated in the pluriallelic restriction of peptide-specific T-cell lines. *Hum Immunol* **40**: 279–290

McKinney JS, Fu XT, Swearingen C, Klohe E and Karr RW (1994) Individual effects of the DR11-variable beta-chain residues 67, 71, and 86 upon DR (alpha, betal*1101)-restricted, peptide-specific-T cell proliferation. *J Immunol* **153**: 5564–5571

Pardoll DM and Topalian SL (1998) The role of CD4+ T cell responses in anti tumor immunity. *Curr Opin Immunol* **10**: 588–594

Peoples GE, Yoshino I, Douville CC, Andrews JV, Goedegebuure PS and Eberlein TJ (1994) TCR Vbeta3+ and V beta6+ CTL recognize tumor-associated antigens related to HER2/neu expression in HLA-A2+ ovarian cancers. *J Immunol* **152**: 4993–4999

Peoples GE, Goedegebuure PS, Smith R, Linehan DC, Yoshino I and Eberlein TJ (1995) Breast and ovarian cancer-specific cytotoxic T lymphocytes recognize the same HER2/neu-derived peptide. *Proc Natl Acad USA* **92**: 432–436

Reichert A, Heitz D, Echner H, Voelter W and Faulstich H (1996) Identification of contact sites in the actin-thymosin β4 complex by distance-dependent thiol cross-linking. *J Biol Chem* **271**: 1301–1308

Ridge JP, Di Rosa F and Matzinger P (1998) A conditioned dendritic cell can be temporal bridge between a CD4+ T-helper and a T-killer cell. *Nature* **393**: 474–478

Rongcun Y, Salazar-Onfray F, Charo J, Malmberg KJ, Evrin K, Maes H, Kono K, Hising C, Petersson M, Larsson O, Lan L, Appella E, Sette A, Celis E and Kiessling R (1999) Identification of new HER2/neu-derived peptide epitopes that can elicit specific CTL against autologous and allogeneic carcinomas and melanomas. *J Immunol* **163**: 1037–1044

Schoenberger SP, Toes RE, Van Der Voort EI, Offringa R and Melief CJ (1998) T-cell help for cytotoxic T lymphocytes is mediated CD40-CD40L interactions. *Nature* **393**: 480–483

Schultz LB and Weber BL (1999) Recent advances in breast cancer biology. *Curr Opin Oncol* **11**: 429–434

Simpson E, Gordon RD (1997) Responsiveness to H-Y Antigen, Ir-gene complementation and target specificity. *Immunol Rev* **35**: 59–75

Southwood S, Sidney J, Kondo A, Del Guercio MF, Appella E, Hoffman S, Kubo RT, Chesnut RW, Grey HM and Sette A (1998) Several common HLA-DR types share largely overlapping peptide binding repertoires. *J Immunol* **160**: 3363–3373

Topalian SL, Gonzales MI, Parkhurst M, Li YF, Southwood S, Sette A and Rosenberg SA (1996) Melanoma-specific CD4+ T cells recognize nonmutated HLA-DR-restricted tyrosinase epitopes. *J Exp Med* **183**: 1965–1971

Tuttle TM, Anderson BW, Thompson WE, Lee JE, Sahin A, Smith TL, Grabstein KH, Wharton JT, Ioannides CG and Murray JL (1998) Proliferative and cytokine responses to class II HER-2/neu-associated peptides in breast cancer patients. *Clin Cancer Res* **4**: 2015–2024

Wang RF and Rosenberg SA (1999) Human tumor antigens for cancer vaccine development. *Immunol Rev* **170**: 85–100

Yoshino I, Goedegebuure PS, Peoples GE, Parikh AS, Dimaio JM, Lyerly HK, Gazdar AF and Eberlein TJ (1994) HER2/neu-derived peptides are shared antigens among human non-small cell lung cancer and ovarian cancer. *Cancer Res* **54**: 3387–3390

Zeliszewski D, Golvano JJ, Gaudebout P, Dorval I, Freidel C, Gebuhrer L, Betuel H, Borras-Cuesta F and Sterkers G (1993) Implication of HLA-DR residues at positions 67, 71, and 86 in interaction between HLA-DR11 and peptide HA306-320. *J Immunol* **151**: 6237–6247

*© 2001 Cancer Research Campaign*
